# Tumor image-derived texture features are associated with CD3 T-cell infiltration status in glioblastoma

**DOI:** 10.18632/oncotarget.20643

**Published:** 2017-09-05

**Authors:** Shivali Narang, Donnie Kim, Sathvik Aithala, Amy B. Heimberger, Salmaan Ahmed, Dinesh Rao, Ganesh Rao, Arvind Rao

**Affiliations:** ^1^ Department of Bioinformatics and Computational Biology, The University of Texas MD Anderson Cancer Center, Houston 77030, TX, USA; ^2^ Department of Neurosurgery, The University of Texas MD Anderson Cancer Center, Houston 77030, TX, USA; ^3^ Department of Diagnostic Radiology, The University of Texas MD Anderson Cancer Center, Houston 77030, TX, USA; ^4^ Department of Radiology, The University of Florida College of Medicine, Jacksonville 32209, Florida, USA; ^5^ Department of Radiation Oncology, The University of Texas MD Anderson Cancer Center, Houston 77030, TX, USA

**Keywords:** imaging-genomics analysis, texture analysis, immune activity, glioblastoma

## Abstract

This study analyzed magnetic resonance imaging (MRI) scans of Glioblastoma (GB) patients to develop an imaging-derived predictive model for assessing the extent of intratumoral CD3 T-cell infiltration. Pre-surgical T1-weighted post-contrast and T2-weighted Fluid-Attenuated-Inversion-Recovery (FLAIR) MRI scans, with corresponding mRNA expression of CD3D/E/G were obtained through The Cancer Genome Atlas (TCGA) for 79 GB patients. The tumor region was contoured and 86 image-derived features were extracted across the T1-post contrast and FLAIR images. Six imaging features—kurtosis, contrast, small zone size emphasis, low gray level zone size emphasis, high gray level zone size emphasis, small zone high gray level emphasis—were found associated with CD3 activity and used to build a predictive model for CD3 infiltration in an independent data set of 69 GB patients (using a 50-50 split for training and testing). For the training set, the image-based prediction model for CD3 infiltration achieved accuracy of 97.1% and area under the curve (AUC) of 0.993. For the test set, the model achieved accuracy of 76.5% and AUC of 0.847. This suggests a relationship between image-derived textural features and CD3 T-cell infiltration enabling the non-invasive inference of intratumoral CD3 T-cell infiltration in GB patients, with potential value for the radiological assessment of response to immune therapeutics.

## INTRODUCTION

The presence and nature of the intratumoral immune response has been shown to influence tumor progression and prognosis [1], including in glioblastoma (GB), the most common primary brain tumor in humans [2–4]. Although a variety of markers have been used for assessing an intratumoral immune response, CD3 (cluster of differentiation 3) is one such reliable marker. CD3 is a protein complex composed of one chain of CD3D, one chain of CD3G, and two chains of CD3E, and is a general marker of T-cells. Robust antitumor immune responses have been shown to correlate with clinical responses to a variety of immune therapeutics [5–7], including tumor-infiltrating CD3 T-cells within the context of a dendritic cell therapy for GB patients [7]. Immune therapeutics are demonstrating promising response rates in GB patients despite prior notions that the central nervous system (CNS) is “immune privileged”. Determination of intratumoral immune influx and/or response to immune therapeutics currently requires either biopsy or surgery, with their inherent risks and sampling limitations.

A large body of work to date has examined the relationships between tumor-derived phenotypic features (tumor volume, heterogeneity etc.) and tumor genetics. For example, statistical summary measurements derived from tumor intensity histograms have been shown to be associated with KRAS mutation status in colorectal tumors [8] and non-small cell lung cancers [9], as well as pathology/molecular grade in gliomas [10]. These features are related with the use of feature ratios across spatial scale, derived from Laplacian-of-Gaussian filtered (PET/CT, CT respectively) images. Other studies have focused on transform-domain characterization (such as the S-transform) to measure association of tumor texture with p53 status in head and neck tumors [11]. Such characterization has proven useful for gliomas as well, for example in the assessment of MGMT methylation status [12], 1p/19q co-deletion in oligodendrogliomas [13] and IDH mutation status [14]. Additionally, other studies have focused on the predictive value of tumor volumetrics to characterize the molecular subtypes in GB [15, 16]. Furthermore, for GB as well as other tumors, a significant amount of work has aimed to investigate the relationship between tumor image-derived features and gene expression programs [10, 17–20]. Gray level co-occurrence based texture features extracted at multiple spatial scales (using wavelets etc.) have also been found to be relevant for understanding disease biology for lung tumors [21], head and neck tumors [22], and breast tumors [23]. Texture analyses using modalities such as MRI, positron emission tomography (PET), and computed tomography (CT) have all been shown to be promising for characterizing cancer genetics [24–27].

Relationships between image-derived features and intratumoral immune responses may enable the noninvasive inference of the immune status of patients for whom imaging is central to the diagnosis, monitoring and evaluation of response to immune therapeutics. Significant progress has been made in understanding the genetic alterations in GB [28, 29]. For GB, assessment of tumor molecular status using image-derived features has been explored [15, 16, 20], but such investigations for the noninvasive assessment of the presence of an immune response have not been described. The ability to predict intratumoral immune reactivity (specifically, CD3 activity) in GB is relevant, as a recent study proposed that the ratio of tumor volume in the T2-FLAIR scan relative to the volume of contrast enhancement was associated with the mesenchymal subtype of GB [16]. This subtype of GB has been previously shown to have a more robust immunological response relative to the other three GB subtypes [30]. Such exploration could potentially be helpful to assess the response of immune therapeutics for GB patients based on a noninvasive assessment of tumor immunological status. With this goal, we sought to develop an approach to assess the relationship between the CD3 T-cell infiltration status within GBs and image-derived gray level heterogeneity features.

## RESULTS

### Patients

A set of glioblastoma (GB) 79 patients were selected from the TCGA database based on quality assessments, as well as the availability of T1-weighted-post-contrast, T2-FLAIR images and accompanying clinical and molecular data, specifically CD3D/E/G mRNA expression level data. Following image-preprocessing, tumor segmentation and analysis of T1w and T2-FLAIR images, 86 imaging (radiomic) features were obtained. Boruta feature selection [32] was then used to identify a set of 6 CD3-associated image features. An image-based predictive model (based on these six features) for CD3 infiltration status was constructed on a secondary cohort of 69 GB patients treated at MD Anderson. Half of the MD Anderson cohort was used as the training set (i.e., for model construction), and the other half was used for model evaluation. The Kruskal-Wallis test is used to assess that the clinical and tumor volume/intensity characteristics between the training and testing sets are similar (none of the p-values are significant at the 0.05 level).

### MRI features are associated with CD3 activity

Among the 86 MRI features, 6 of them are found to be associated with CD3 mRNA expression (after minimizing inter-feature correlation). These are, kurtosis, contrast, small zone size emphasis, low gray-level zone emphasis, high gray-level zone emphasis, and small zone high gray emphasis.

### Imaging feature-based model is capable of predicting CD3 T-Cell infiltration status

**Figure 1:**
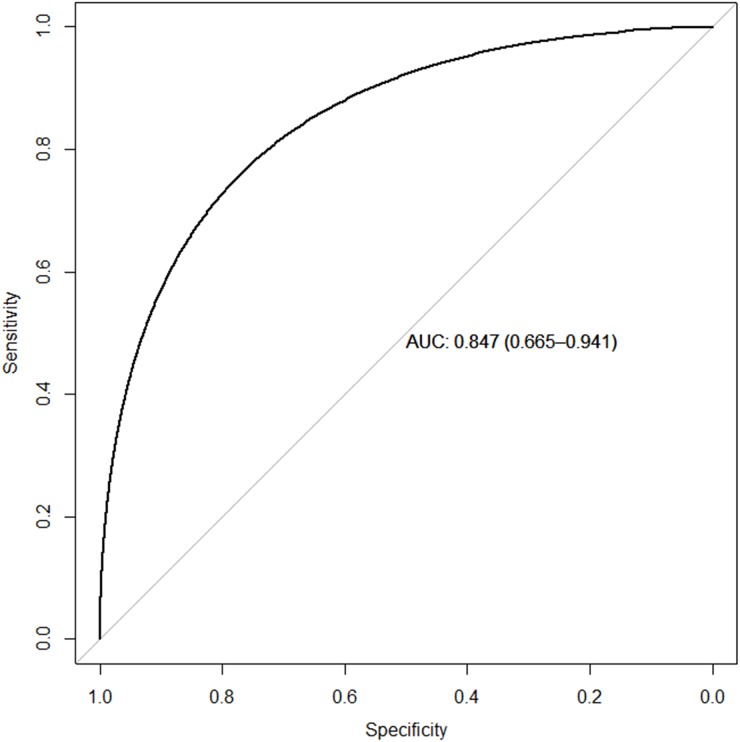
Receiver operating curve for the prediction of CD3 T-cell infiltration Half of internal cohort is considered as a training set (for model construction) and the remaining half of the internal cohort is considered as a testing set (for model evaluation).

In the testing cohort, the performance of the model for prediction of CD3 T-cell infiltration was an AUC of 0.847 with confidence interval (CI) of [0.66, 0.94] (Figure 1). Spearman's rank correlation coefficient between model-predictions and actual CD3 counts was 0.544 (p-value of 0.0009). The confusion matrix (Table 1) showed an accuracy of 76.5% with 95% CI of 0.588-0.893. The model has 72.7% sensitivity, 83.33% specificity, and 11.1% false discovery rate (FDR). The corresponding AUC on the training cohort was 0.993 (97.1% accuracy, 93.75% sensitivity, 100% specificity, and 0% FDR). To assess the significance of the image model-derived predictions for CD3 infiltration status after adjusting for clinical variables and tumor volumetrics, a multivariate regression was performed. The result of the multivariate regression approach is summarized in Table 2, and only the model-derived predictions are seen to be significant (p-value of 0.03). In addition, a comparison of predictive performance (AUC) of a model based only on tumor volumetrics (from T1-post contrast and T2-FLAIR imaging) relative to a combined model (incorporating both volumetrics and texture features) revealed AUCs of 0.73 and 0.92 respectively (p-value:0.047).

**Table 1:** Confusion matrix for both training and testing cohorts

Training			
		Actual	
		Low Infiltration	High Infiltration
Prediction	Low Infiltration	19	1
	High Infiltration	0	15
**Testing**			
		**Actual**	
		**Low Infiltration**	**High Infiltration**
Prediction	Low Infiltration	10	6
	High Infiltration	2	16

**Table 2:** Summary of multivariate regression to assess relationship between image-derived prediction model and CD3 activity, after adjusting for various clinical covariates (age, KPS, gender, total intensity, and tumor volume). The p-values of each term in the multivariate model are indicated below

Variables	p-value
Age	0.5591
Gender	0.2842
KPS	0.7997
T2-FLAIR Tumor Volume	0.8775
T2-FLAIR total intensity	0.8077
T1-Post Tumor Volume	0.8301
T1-Post total intensity	0.5701
Predicted Values from our model	0.0309

### Comparison of individual feature performance vs. combined model for predicting CD3 status

Table 3 summarizes the individual feature performance for modeling the binary CD3 T-cell response. Small zone high gray emphasis is the individual image feature with the highest AUC value (0.79) in the testing cohort, although lower when compared to the overall model obtained from combining all the six features together (AUC = 0.847).

**Table 3:** Summary of CD3-associated image features and performance of the CD3 prediction model (based on individual features and also of the feature combination) in the testing cohort

Modality	Feature type	Feature	AUC	95% confidence interval
T1 Post	Histogram	Kurtosis	0.736	0.517-0.885
T1 Post	NGTDM	Contrast	0.759	0.557-0.891
T1 Post	GLSZM	Small Zone Size Emphasis	0.72	0.521-0.870
T1 Post	GLSZM	Low gray-level zone emphasis	0.673	0.468-0.860
T1 Post	GLSZM	High gray-level zone emphasis	0.745	0.568-0.894
T1 Post	GLSZM	Small zone high gray emphasis	0.798	0.619-0.911
All combined			0.847	0.66 -0.94

Abbreviations: NGTDM, Neighborhood Gray Tone Difference Matrix; GLSZM, Gray Level Size Zone Matrix

## DISCUSSION

In this study, we demonstrated that MR image-derived textural features have predictive value for assessment of CD3 activity within GB. Among the six image-based textural features used for model construction, 4 are GLSZM-derived (Gray Level Size Zone Matrix [33]) features while the other two are intensity histogram-derived (kurtosis) and NGTDM-derived (Neighborhood Gray Tone Difference Matrix [34]) (contrast) features. The kurtosis/ contrast features highlight ring-like patterns of enhancement as well as intensity variations at the tumor boundary. On the other hand, in GLSZM, the entry (i, j) represents the number of 3D zones of gray levels *i* and of size *j* [33]. This matrix essentially captures coarse texture based on the observation that a relatively large zone size occurs more often in a coarse texture than in a smooth one [35]. Considering GB tumors exhibit large variations in intensity, this observation suggests that GLSZM-derived features are potentially effective statistical representations of the tumor MRI image for assessment of CD3 infiltration. In broader context, the increased immune influx increases tumor heterogeneity features [31] which are likely reflected in image-derived textural diversity [36]. These features are related to variation [37] in extent of contrast enhancement within the tumor possibly related to changes [38] in vascularization and inflammatory status [39]. However, a systematic perturbation approach, possibly in an animal model, may clarify the nature of relationship between CD3 infiltration status and these radiographic phenotypes.

The model achieved an AUC of 0.847 for the prediction of CD3 T-cell infiltration in the testing cohort (Figure 1). To assess that its predictive value is not confounded by clinical factors or tumor volume, a multivariate regression model reveals the association of CD3 infiltration status with image-based, model-derived predictions (p-value of 0.0309, Table 2). In addition, a model combining imaging and volumetrics has a superior AUC (0.847) relative to one with a volumetrics-only model (AUC 0.73). However, a study with a larger sample size could further clarify the predictive value (e.g. a narrower confidence interval for the AUC) of this image-derived radiomic model.

This study suggests the potential value of MRI-derived texture features for assessing the inflammatory status within a tumor. While additional and larger-scale validation studies would enable a clearer assessment of their clinical value, textural MRI analysis may be useful in stratifying patients for therapeutics that require a pre-existing immune response [40, 41] to longitudinally monitor the kinetics of antitumor immune responses, to adjust treatment scheduling, and to obtain early assessments of responding patient subsets. This approach could also be potentially useful in low-resource healthcare settings where invasive genetic assessments may not be accessible or affordable. The textural MRI analysis will be prospectively validated in an immune inhibitory checkpoint clinical trial in patients with recurrent GB, which will correlate *ex vivo* flow cytometry quantification of the absolute number and functional status of T-cells (NCT02337686).

Our study presents several opportunities for future work. Because this study focuses on texture features derived only from T1-post and T2-FLAIR sequences, a suitable next step would be the investigation of other sequences such as multi-echo magnetization-prepared rapid gradient-echo (MEMPRAGE) or Diffusion Weighted Intensity MRI, to assess their added value. Further, as with any retrospective analysis of multi-site radiology data, a limitation in this study is the variation in scanning and acquisition protocols across MRI systems and imaging sites within the publicly available TCIA database. While preprocessing (isotropic reslicing [42] and intensity normalization) was performed to account for such variation, their influence (of variable intensity characteristics, differing spatial resolutions etc.) on image-derived textural features needs to be examined more systematically. Another avenue for investigation is the impact of intensity normalization approaches [43] and varying gray levels (4, 16, 32, etc.) on texture features and their predictive accuracy for CD3 activity. Also, the current prediction model is based on a (binary) dichotomization of the CD3 activity status. This was done to guard against the subjectivity associated with human-scored measurements of CD3 infiltration. However, the use of more automated methods of infiltration-assessment may allow direct use of continuous-valued CD3 T-cell counts within regression models.

To further assess the generalizability of the model's predictive capabilities, a measurement of the relative change in texture features under the test-retest setting [44, 45] would strengthen its path to clinical adoption. Further, other independent data sets with consistent imaging protocols accompanied by adjustment for molecular status (IDH1, EGFR mutation etc) would enable a more robust assessment of predictive value of this image-derived radiomic model. Also, since the current model is based solely on pre-therapy, pre-surgical images, another aspect would be to study the longitudinal variations in texture characteristics during the course of therapy. Such a study might illuminate a potential path to assessing therapy-induced changes via texture measurements and its feasibility in the context of determining the presence of intratumoral inflammation during the course of therapy.

## MATERIALS AND METHODS

### Patient selection

For this study, we used imaging and genomic data from 79 patients with histologically confirmed GB and molecular information in TCGA database. Patients were selected based on quality assessments by the TCGA Glioma Phenotype Research group, in addition to having high quality T1-weighted post-contrast and T2-FLAIR images with accompanying clinical information. The entire case-list of patients is presented in Table 1 of the Supplementary data. Pre-surgical, post-contrast axial T1-weighted and axial T2-weighted FLAIR images for these 79 patients were obtained from The Cancer Imaging Archive (https://www.cancerimagingarchive.net/). The TCIA voxel dimensions range 0.469 ~ 1.016mm, 0.469 ~ 1.016mm, and 0.700 ~ 6.500 mm for x, y, and z directions respectively (Supplementary Information 2). CD3D/E/G mRNA expression z-scores were obtained from the MSKCC cBioPortal (https://www.cbioportal.org) for these 79 TCGA patients. This dataset was used for identifying CD3-associated image features. Under TCGA data-use agreements, this portion of the study was exempt from Institutional Review Board approval. A separate data set of 69 GB patients (from MD Anderson) in which CD3 cell counts were obtained by immunohistochemistry [3] (under the Institutional Review Board-approved protocol LAB03-0228), designated as the “MD Anderson cohort”, was used to obtain an image-based predictive model of CD3 T-cell infiltration, using half the cohort for model development and the other half for model evaluation. The voxel sizes for MD Anderson Cohort data range from 0.430 ~ 1.016mm, 0.430 ~ 1.016mm, 1.800 ~ 7.501mm for x, y, and z directions respectively (Supplementary Information 2). The clinical data characteristics, including age, Karnofsky Performance Score (KPS), tumor volume, and sex for both the TCGA and the MD Anderson cohort are also summarized in Table 4.

**Table 4:** Summary of clinical covariates for TCGA and the internal (MD Anderson) data cohort. Additionally, p-values comparing the distributions of these clinical variables between the two cohorts are shown below (p-values are not significant, suggesting that the TCGA and internal cohorts are comparable)

	TCGA GBM cohort (mean + std. dev)	IHC data cohort (MD Anderson Cancer Center) (mean +std. dev)	p-values
Age	56.84 + 14.96	57.96 + 13.78	0.638
Gender	F – 26; M – 53	F – 33; M – 36	0.105
Karnofsky Performance Score (KPS)	81.01 + 11.86	84.64 + 14.4	0.102
FLAIR tumor volume (in mm3)	130761.35 + 74839.78	142820.01 + 89254.72	0.381
T1 tumor volume (in mm3)	51677.66 + 32966.17	52738.99 + 38800.02	0.86
Number of Subjects	79	69	

### Image preprocessing

MR images from both internal study (MDACC) patients and public domain TCGA cases were processed prior to tumor segmentation. Specifically, the T1-post contrast and T2-FLAIR images were corrected for shading artifacts with non-parametric intensity non-uniformity normalization (N3) correction using Medical Image Processing Analysis and Visualization software (v 7.2.0) [46]. For this procedure, the signal threshold value was 1.0, field distance was 50.0 mm, and kernel full width half max (FWHM) was 0.15.

### Volumetric segmentation

**Figure 2:**
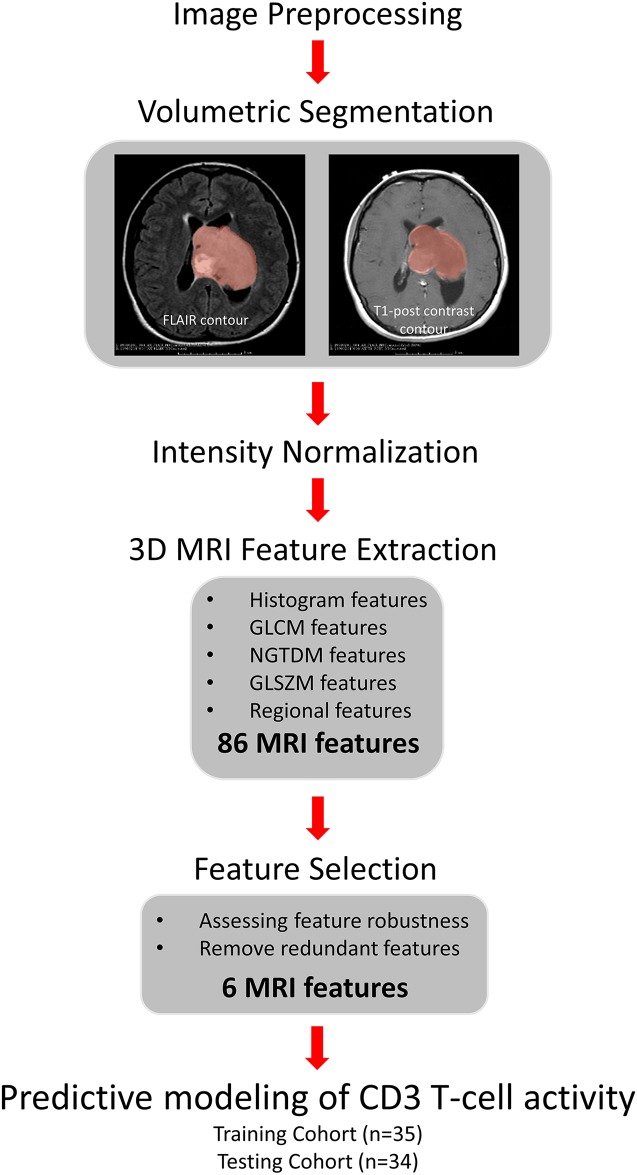
The pipeline for feature extraction and predictive modeling We contoured tumor regions from pre-processed T1-post and T2-FLAIR images and performed intensity normalization. 86 MRI image features were derived from TCGA data set and reduced to 6 by robustness analysis and redundant feature removal. Using these 6 features, we constructed a predictive model for CD3 activity using the training set, and evaluated on the testing set.

Tumors were segmented (contoured) semi-automatically in 3D using the Medical Image Interaction Toolkit –MITK3M3 Image Analysis (v 1.1.0) (www.mint-medical.de/productssolutions/mitk3m3). This software has been validated as a method to segment tumors in various organ systems [47, 48]. The clinician uses the segmentation tool to contour the relevant regions (enhancing tumor on the T1-post contrast images, hyperintensity on the FLAIR images) on multiple slices and then interpolates those contours to obtain the 3D volumetric tumor mask. On the T1-post contrast images, the segmented region corresponds to the contrast enhancing tumor. For the T2-FLAIR images, the segmented region corresponds to the solid tumor as well as regions of infiltrating tumor and edema that are delineated by increased intensity. The semi-automatic segmentation tools provided by MITK3M3 permit the discrimination of tumor from normal surrounding brain. In addition, using a similar approach, normal appearing white matter area in the contralateral region was contoured separately on both T1-post contrast and T2-FLAIR images. The same set of preprocessing and segmentation procedures was followed on both (TCGA/MD Anderson) data cohorts. Segmentation masks were reviewed for accuracy by two fellowship-trained neuro-radiologists (SA and DR). The entire workflow along with description for each step is shown in Figure 2.

### Image feature extraction

Tumor heterogeneity characteristics from the image are measured using computer-based textural analysis. First, tumor region intensities were normalized relative to the mean intensity value of normal appearing white matter (NAWM) region. Images were resliced for pixel size (1×1×1 mm) using the NIFTI toolbox [49], to make voxel definitions consistent across the multiple sites contributing imaging data to TCGA/TCIA. The intensities of resliced images were quantized [50] to 8 gray levels prior to computation of texture features.

Once scaled and quantized, we used a public domain MATLAB radiomics toolbox [51] to extract 3D tumor derived features: 6 histogram, 19 Gray Tone Spatial Dependence Matrix (GTSDM), 5 Neighborhood Gray Tone Difference Matrix (NGTDM), and 11 Gray Level Size Zone Matrix (GLSZM) based features (41 features in total) were computed from both the T1 post-contrast and FLAIR images. In addition, two additional (regional) features, sum of the normalized tumor intensities as well as tumor volume, were computed. In total, we obtained 86 features, 43 each from the T1-post contrast and T2-FLAIR images, respectively. All the procedures were performed in Windows 7 environment (Intel ® Xeon ® X5570 CPU @ 2.93 GHz and 32.0 GB RAM).

### Assessment of robustness of image features

The dimensions of the final TCGA image-feature data matrices were 79 × 86 (79 patients, 86 image-derived features). For the internal cohort (corresponding to CD3 IHC-derived cell count measurements), the matrix dimensions were 69 × 86 (69 patients in the internal MDACC data). Following prior work [18], 8 different geometric perturbations were applied to the tumor ROIs (both T1-post contrast and T2-FLAIR) to assess feature robustness using the intra-class correlation (ICC). These transformations include horizontal translation by 2 pixels, horizontal and vertical translation by 2 pixels, 1-degree rotation, 5-degree rotation, moving each point on the ROI outline by a random value with (zero-mean and 0.1 and 0.5-pixel standard deviation), enlarging by 1 pixel radially, and shrinking by 1 pixel radially [18]. Intra-class correlation coefficient of 0.6 [18] was used to identify features that are robust between the original ROI and the geometrically-transformed versions.

### Feature selection: identification of features associated with z-score transformed CD3 mRNA activity, using TCGA cohort

Using the robust features identified with the above-mentioned approach, the R Boruta package [32] is used for feature selection, in order to identify image features associated with CD3E/D/G mRNA expression z-scores in the TCGA dataset. With a relatively large number of image features, it is necessary to identify the subset of features that are both non-redundant and “relevant” to the outcome. The Boruta algorithm allows one to perform “all-relevant” feature selection [32]. Instead of only identifying the minimal feature subset to fit the data, it is capable of identifying ‘every’ feature (under some restrictions) associated with the outcome, (the CD3 mRNA expression z-scores). The algorithm works by assessing the importance of a feature relative to ‘shadowed’ versions (obtained by permuting its values across cases) and only retaining those features with importance more significant than their shadowed version. Subsequently, the CD3 mRNA-associated image features were reduced using inter-feature correlations thresholded at (absolute value of) 0.6. These retained features were then used for classifier construction.

### Determination of CD3 infiltration status based on image-derived features: model construction and evaluation

For the internal (MDACC) cohort, CD3 cell count z-scores were dichotomized (relative to 0) yielding low or high CD count classes. These are treated as (binary) class labels. A randomly chosen subset (of half the cases) was used as the training set to construct the model, while the other half was used as the test set. These two groups were ensured to have similar clinical and intensity/volume characteristics based on Kruskal-Wallis test (Table 4). A prediction model using symbolic regression [52] was constructed based on the training set, using the six features identified based on the TCGA data.

### Statistical analysis

All statistical analyses were performed using R 2.15.3. Using the ground truth class labels, we computed the receiver operating characteristic (ROC) curve using model predictions. Confidence intervals of the ROC area-under-curve (AUC) relative to random classification was computed via stratified bootstrap sampling [52]. We also assessed the concordance between the actual CD3 count values and model predictions, using Spearman's rank correlation analysis. In order to ensure that the clinical and tumor volume/intensity characteristics of the training and testing sets are comparable, for consistency during model construction and evaluation, we performed Kruskal-Wallis test between the two sets (Table 4). Further, to assess whether the model performance is confounded by clinical and tumor volume/intensity characteristics, we performed a multivariate regression to determine the relationship between the model predictions and the ground truth CD3 measurements after adjusting for these clinical covariates. We also evaluated the difference in AUCs of model involving only tumor volumetrics (from T1-post contrast and T2-FLAIR) relative to a model involving both volumetrics and image-derived texture features, using a bootstrap based test [53]. Lastly, in order to assess the predictive performance of the 6 image features individually, we constructed six separate models for CD3 prediction based on the 6 features and compared them against the combined 6 feature-model on the testing set.

## SUPPLEMENTARY MATERIALS FIGURES AND TABLES




