# Supplementary material for: Tumor image-derived texture features are associated with CD3 T-cell infiltration status in glioblastoma
Source: Oncotarget. 2017 Sep 5;8(60):101244–54. doi: 10.18632/oncotarget.20643 (PMC5731870; doi:10.18632/oncotarget.20643)
Supplement: Supplementary file 1 [file oncotarget-08-101244-s001.pdf]

Tumor image-derived texture features are associated with CD3 T-cell infiltration status in glioblastoma

SUPPLEMENTARY MATERIALS

Supplementary Table 1: TCGA patient list used for this study

See Supplementary File 1

Supplementary Table 2: Range in Voxel dimensions (in mm) along x, y, and z directions for TCGA and Internal patients

	TCGA			MD Anderson		
	x	y	z	x	y	z
min	0.469	0.469	0.700	0.430	0.430	1.800
max	1.016	1.016	6.500	0.977	0.977	7.501
