# Supplementary material for: Tumor image-derived texture features are associated with CD3 T-cell infiltration status in glioblastoma
Source: Oncotarget. 2017 Sep 5;8(60):101244–54. doi: 10.18632/oncotarget.20643 (PMC5731870; doi:10.18632/oncotarget.20643)
Supplement: Supplementary file 2 [file oncotarget-08-101244-s002.docx]

**Supplementary information**

**Table 1: TCGA patient list used for this study:**

| **Case-ids** |
| --- |
| TCGA-02-0011 |
| TCGA-02-0027 |
| TCGA-02-0033 |
| TCGA-02-0034 |
| TCGA-02-0046 |
| TCGA-02-0047 |
| TCGA-02-0060 |
| TCGA-02-0064 |
| TCGA-02-0068 |
| TCGA-02-0069 |
| TCGA-02-0070 |
| TCGA-02-0075 |
| TCGA-02-0085 |
| TCGA-02-0086 |
| TCGA-02-0087 |
| TCGA-02-0102 |
| TCGA-02-0106 |
| TCGA-06-0122 |
| TCGA-06-0127 |
| TCGA-06-0129 |
| TCGA-06-0132 |
| TCGA-06-0133 |
| TCGA-06-0137 |
| TCGA-06-0138 |
| TCGA-06-0139 |
| TCGA-06-0145 |
| TCGA-06-0147 |
| TCGA-06-0148 |
| TCGA-06-0149 |
| TCGA-06-0154 |
| TCGA-06-0158 |
| TCGA-06-0162 |
| TCGA-06-0164 |
| TCGA-06-0166 |
| TCGA-06-0168 |
| TCGA-06-0171 |
| TCGA-06-0173 |
| TCGA-06-0174 |
| TCGA-06-0175 |
| TCGA-06-0176 |
| TCGA-06-0177 |
| TCGA-06-0179 |
| TCGA-06-0184 |
| TCGA-06-0185 |
| TCGA-06-0187 |
| TCGA-06-0188 |
| TCGA-06-0189 |
| TCGA-06-0190 |
| TCGA-06-0210 |
| TCGA-06-0237 |
| TCGA-06-0238 |
| TCGA-06-0241 |
| TCGA-06-0644 |
| TCGA-06-0646 |
| TCGA-08-0244 |
| TCGA-08-0246 |
| TCGA-08-0348 |
| TCGA-08-0350 |
| TCGA-08-0352 |
| TCGA-08-0353 |
| TCGA-08-0354 |
| TCGA-08-0355 |
| TCGA-08-0356 |
| TCGA-08-0357 |
| TCGA-08-0358 |
| TCGA-08-0359 |
| TCGA-08-0360 |
| TCGA-08-0385 |
| TCGA-08-0389 |
| TCGA-08-0390 |
| TCGA-08-0392 |
| TCGA-08-0509 |
| TCGA-08-0510 |
| TCGA-08-0512 |
| TCGA-08-0518 |
| TCGA-08-0520 |
| TCGA-08-0521 |
| TCGA-08-0524 |
| TCGA-08-0529 |
